# A Non-Invasive Physiological Control System of a Rotary Blood Pump Based on Preload Sensitivity: Use of Frank–Starling-Like Mechanism

**DOI:** 10.3390/mi13111981

**Published:** 2022-11-15

**Authors:** Fangqun Wang, Shaojun Wang, Zhijian Li, Chenyang He, Fan Xu, Teng Jing

**Affiliations:** 1 School of Electrical and Information Engineering, Jiangsu University, Zhenjiang 212013, China; 2 National Research Center of Fluid Machinery Engineering and Technology, Jiangsu University, Zhenjiang 212013, China

**Keywords:** rotating blood pumps, non-invasive control system, Frank–Starling-like mechanism, preload sensitivity

## Abstract

Implanting rotary blood pumps (RBPs) has become the principal treatment for patients suffering from severe heart failure. There are still many challenges to address for RBP control systems. These problems include meeting the patient’s physiological perfusion, eliminating postoperative complications, as well as debugging the patient’s physiological control system (automatically and indiscriminately). This paper proposes a non-invasive adaptive control system based on the Frank–Starling-like mechanism (NAC-FSL) to solve these problems. This control system uses the motor speed of the rotary blood pump as the only input variable, and the pump flow was estimated by the motor speed for achieving non-invasive detection. Simultaneously, a cardiovascular reference model was developed to provide an appropriate real-time preload for heart failure patients. The Frank–Starling-like control baseline was tracked to obtain the desired reference average pump flow by using the preload. Avoiding suction was done by adopting the control baseline (CLn), which included a flat slope under a high preload. Moreover, the NAC-FSL system could potentially unload the left ventricle and provide a higher pump flow with a smaller error during the exercise state, as compared to the CSC system. Finally, the K value indicating the preload sensitivity in the NAC-FSL controller was optimized to meet the perfusion needs according to the hemodynamic parameters.

## 1. Introduction

RBPs acting as left ventricular assist devices (LVADs) always operate in a constant-speed mode in clinics, providing sufficient output for serious heart failure patients. Although this control method possesses the merits of simplicity, reliability, stability, and durability, it reduces pulsatility, which may lead to a variety of complications [1,2]. Moreover, this control mode cannot adapt to a patient’s complex and variable physiological environment. Owing to the lower preload sensitivity of RBPs in the conventional constant speed controller (CSC) [3], various physiological control techniques have been developed to match the pump output to physiological perfusion requirements [4].

To meet the perfusion prerequisites for different physiological states of the patient, a variable speed physiological control system is needed for adjusting the motor speed. The Frank–Starling mechanism that mimics the natural heart has been used widely in the development of variable speed physiological control systems [5,6,7,8,9]. Stevens MC et al. proposed a control strategy mimicking the Frank–Starling mechanism by directly measuring LAP with a pressure sensor and mounting a flow sensor at the pump outlet catheter used to measure pump flow [5]. The control system adjusts the pump speed according to the measured pump flow rate, and the Frank–Starling mechanism enables the adjusted pump flow rate to correspond to the venous return volume. However, this control strategy relies on pressure and flow sensors. Petrou et al. proposed a physiological controller based on left ventricular systolic pressure [6]. Cordeiroa put forward a synchronous physiological control system of a pulsatile pediatric pump to adjust the pump ejection pressure at each cardiac cycle to keep the mean arterial pressure at a specified reference value [7]. Fetanat developed a novel adaptive physiological control system for an implantable heart pump to respond to interpatient and intra-patient variations to maintain the left ventricular end-diastolic pressure in the normal range to prevent ventricle suction and pulmonary congestion. This study shows that the control performance can be guaranteed across different patients and conditions when using the adaptive physiological control system [8]. Magkoutas presented a physiological data-driven iterative-learning controller (PDD-ILC) that accurately tracked predefined pump flow trajectories, and achieved physiological, pulsatile, and treatment-driven responses of cfVADs [9].

Most physiological control systems use sensors to measure hemodynamic parameters, such as pressure and flow in real time for feedback to the controller. However, there are no commercially available implantable sensors, which are stable over time, and the use of these sensors may increase the risk of postoperative complications, such as thrombosis, or require extensive regulatory inquisition [10]. Therefore, a non-invasive physiological control system using the estimator may solve this problem. Wang Y et al. proposed a sensorless suction prevention and physiologic control (SPPC) algorithm for axial and centrifugal pumps that required only the inherent parameters of the left ventricular assist (pump speed and power) and used a proportional-integral (PI) controller to keep the differential motor speed (△RPM) above a defined threshold [11]. This controller can effectively maintain the mean reference pressure difference between the left ventricle and the aorta to achieve physiological perfusion, but the performance of the algorithm is affected by problems, such as pump thrombosis. Fu and Xu [12] proposed a sensorless fuzzy logic control system that uses motor speed and current as inputs to the system without making invasive measurements. However, the assumption that pump flow is proportional to heart rate ignores the influence of cardiac contractile and peripheral circulation on the required flow. Bakouri [13] used the sliding mode non-invasive control algorithm to take pump speed as the input and pump flow as the estimated output. However, this control algorithm has a corresponding drawback. When left ventricular failure occurs, as is the case in all patients implanted with left ventricular assist devices, the systolic capacity of the left ventricle is severely reduced. Therefore, the dynamic range of the pulsation index is very small, so its ability as a control input is limited. In this paper, a non-invasive adaptive physiological control system based on the Frank–Starling-like mechanism (NAC-FSL) is proposed to solve these problems. The system is also designed to enhance the adaptability to cardiac demand and clinical conditions of the heart that have plagued traditional control strategies. It linked the preload and reference average pump flow and imitated the preload sensitivity of the native heart. This non-linear relationship between the pump flow and ventricular preload enabled the controller to deliver a low preload sensitivity at a high preload, thereby avoiding the ventricle suction and reducing the pump power at a high preload to avoid over-pumping [14,15].

In the preload-based control systems using the Frank–Starling mechanism or Frank–Starling-like mechanism, there are no non-invasive instantaneous preload measurement methods available. Although the preload sensitivity (K) of the left ventricle was primarily defined in a third-order polynomial function by Guyton [16], the determination of the K value, which might affect the robustness of the control system is still unknown. Consequently, this paper will put forward a non-invasive instantaneous preload measurement method and explore the effect of the K value range on the NAC-FSL system under various degrees of heart failure.

## 2. Methodology

### 2.1. Mock Circulation Loop (MCL)

**Figure 1:**
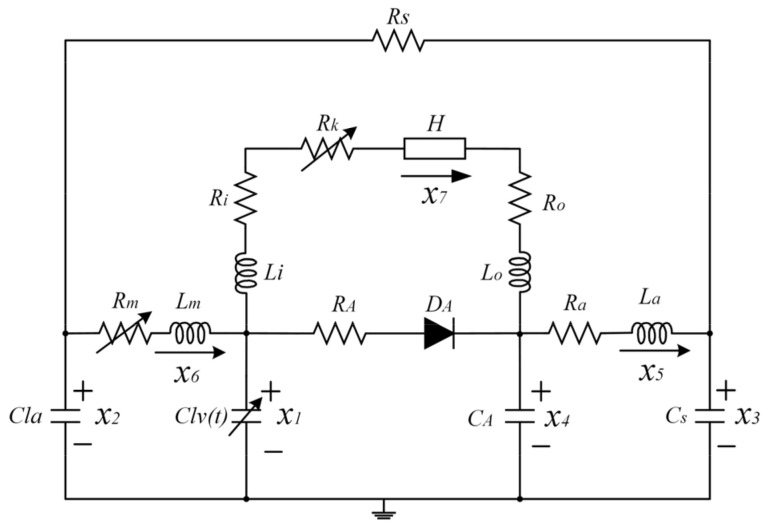
Equivalent circuit model of the LVAD-cardiovascular system.

The classic four-element Windkessel model was used to model the LVAD-cardiovascular system, including a blood pump as LVAD and the vascular system. Figure 1 shows the equivalent circuit model of the system [17]. In this model, Rs is the systemic vascular resistance and can be adjusted according to the patient’s different activity levels, Rm is the time-varying mitral valve resistance, and C_lv_(t) is the ventricular compliance that changes according to left ventricular elasticity. State variables x_1_–x_6_ represent the left ventricular pressure, left atrial pressure, arterial pressure, aortic pressure, aortic flow, and mitral valve flow, respectively, in the cardiovascular system, and x_7_ represents the blood pump flow. Sets of state equations were derived from each of the circuits and combined into simultaneous ordinary differential equations, given as Equation (1), based on state variables. Solving Equation (1), the differential equations with MATLAB R2016a/SIMULINK (the MathWorks, Natick, MA) yields time series data of hemodynamic parameters. For details of parameters, variables, equations, and matrix, refer to reference [16,17].

(1) $$\dot{x}=A\left(t\right)\times x+B\left(t\right)\times p\left(x\right)+C\times u\left(t\right)$$

### 2.2. Control Strategy

**Figure 2:**
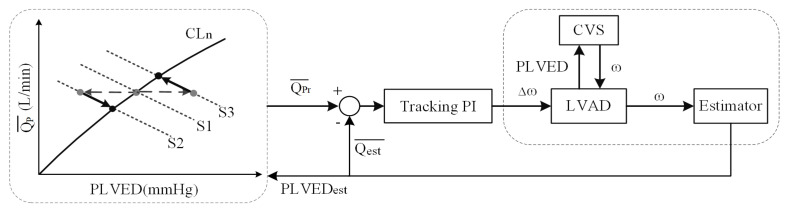
Block diagram of the control system. CVS, reference cardiovascular system; LVAD, left ventricular assist device; ω, motor speed; $\bar{Q_{\mathrm{est}}}$, estimated average pump flow; PLVED, left ventricular end-diastolic pressure; $\bar{Q_{\mathrm{Pr}}}$, desired average pump flow; PI, proportional and integral controller; CLn, Control Line; S1, original state; S2 and S3, deviated states. Gray circles represent the position of operating points after a change in states. Black circles represent the positions of operating points upon arriving at the new steady-state located at the intersection between the control line and the new system line. The controller drives the changes in the operating points along the path indicated by the arrows along the new system line.

A simplified block diagram of the complete control system is represented in Figure 2. Based on the motor speed of the LVAD, a corresponding estimation algorithm was designed to estimate the pump flow ($\bar{Q_{\mathrm{est}}}$) and achieve non-invasive measurement. A cardiovascular model was established to mimic heart failure and provide preload for the control system.

#### 2.2.1. Estimate Average Pump Flow

This paper uses the algorithm proposed by Zhang et al. for measuring the pump flow non-invasively [18]. These parameters were re-fitted by the authors according to the cardiovascular system to achieve better adaptability to this system [17]. The algorithm proved to accurately estimate the pulsating flow and predict regurgitation flow through animal experiments.

In a steady state, the estimated flow $Q_{\mathrm{est}}$ uses the motor’s electric power (Pe) and speed (ω) as inputs, as to the following:

(2) $$Q_{est}(m+1)=a\times Q_{est}(m)-b\times Q_{est}(m-1)+c\times Q_{est}(m-2)+d\times f(m)-e\times f(m-1)$$

(3) $$f(m)=f+g\times Pe(m)+h\times Pe{\left(m\right)}^{2}+i\times Pe{\left(m\right)}^{3}+j\times \omega (m)+k\times \omega {\left(m\right)}^{2}$$

The fitting coefficients a, b, c, d, and e were constants with values 1.98, 1.24, 0.24, 0.27, and 0.25, respectively. Moreover, f, j, and k were constants, and g, h, and i were defined to have a linear relationship with Hematocrit values (HCT). ω was the motor speed, and $Q_{\mathrm{est}}$ was the estimated value of the beat pump flow. f(m) was the input signal of the system. Pe represents electric power, and Q_p_(m) represents the pump flow calculated through state equations to the model developed by the authors in reference [17].

(4) $$Pe(m+1)=\frac{\beta \times \omega^{2}(m)\times Q_{P}(m)}{\delta }$$

(5) $$\delta =\frac{\eta }{\rho \times g}$$

Discrete-time t = m × h, where h was the sampling interval with a 0.0001 value. Meanwhile, the parameter settings are shown in Table 1.

#### 2.2.2. Calculate the Desired Average Pump Flow

To make it easier for doctors in adjusting the reference value for each patient, an adaptive physiological control system was introduced.

A control line was generated using a third-order polynomial function from Equation (6) that fitted into Guyton’s data [16]. This line relates the desired mean pump flow $\bar{Q_{\mathrm{Pr}}}$ to the reference preload (PLVED_m_). A scaling factor (K) was introduced to provide a means of altering the sensitivity of the pump toward the changes in PLVED_m_, which made Equation (6) adaptive to various preload sensitivities of different patients [19]. The range of K values required for patients with different degrees of heart failure will be discussed and studied in the next chapter.

(6) $$\bar{Q_{Pr}}=(0.0003\times PLVED_{m}{}^{3}-0.0276\times PLVED_{m}{}^{2}+0.9315\times PLVED_{m}-0.0928)\times K$$

HR, ω, and Emax are the main three factors affecting PLVED_m_. If Emax is given, PLVED_m_ is estimated through the fitting model (R^2^ > 0.9310) in Equation (7).

(7) $$\begin{array}{l} PLVED_{m}(\omega ,HR)=(1.77\times 10^{-10}\times HR^{2}-1.791\times 10^{-8}\times HR-2.067\times 10^{-7})\times \omega^{2}\\ +(-1.873\times 10^{-5}\times HR+0.00218)\times \omega +1.334\times 10^{-3}\times HR^{2}+0.198\times HR+1.192 \end{array}$$

**Figure 3:**
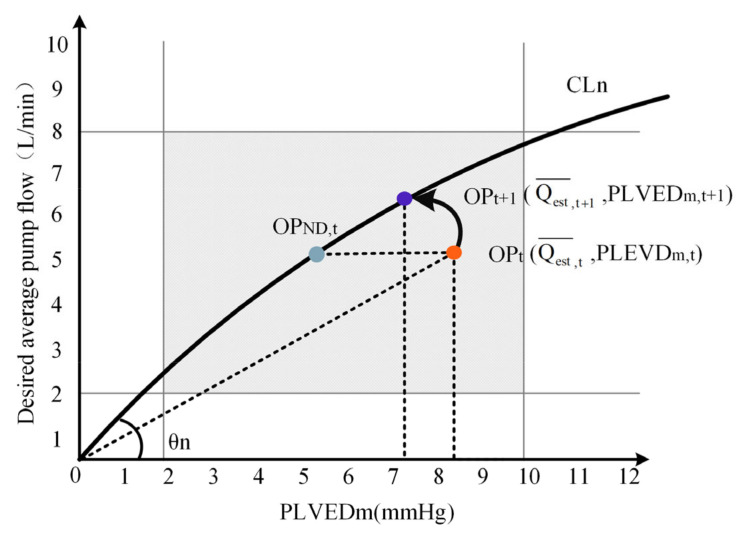
Pump flow regulator. OP_ND,t_, the original operating point; OP_t_, the unadjusted state point of the patient at time t; $\bar{Q_{\mathrm{est},t}}$, the pump flow of the patient at time t; OP_t+1_, the state point after the patient adjusts at t+1; $\bar{Q_{\mathrm{est},t+1}}$, the ideal pump flow after the patient adjusts at time t+1 (reference pump flow); θ_n_, the nth control angle of the operating angle of the baseline.

The preload-based Frank–Starling control system was illustrated in Figure 3. The physiological state of the patient (rest or exercise) was first determined, which was then fed as an input to the reference cardiovascular model, mimicking the heart failure with the same level to obtain a pathological preload. The average pump flow was estimated using the motor speed. The unadjusted state point (OP_t_) was determined according to the preload at t time (PLVEDm). According to Equation (8), the estimated average pump flow returned to CLn to obtain the intersection point OP_t+1,_ which was the appropriate reference point for identifying the patient on the control baseline. The abscissa of OPt+1 was the reference average pump flow ($\bar{Q_{\mathrm{est},t+1}}$) required by the patient [20].

(8) $$\bar{Q_{est,t+1}}=(\sqrt{{\left(\bar{Q_{est,t}}\right)}^{2}+PLVED_{m}{}^{2}})sin\theta_{n}$$

To reduce the risk of abnormal states, such as excessive speed caused by motor failure, suction caused by the failure of the NAC-FSL system, and to provide enough perfusion, the designed average pump flow was limited to between 2 and 8 L/min and the preload was limited to 2–10 mmHg in this paper. The control system works in the gray domain in Figure 3. When the K value is too small, the controller works in an abnormal state.

#### 2.2.3. Motor Speed Control

A PI controller was used to track the error between the actual pump flow and the reference pump flow. Further, the error was used to calculate Δω in Equation (9) to control the motor speed (ω) in each cardiac cycle.

(9) $$\Delta \omega =K_{P}(\bar{Q_{est}}-\bar{Q_{Pr}})+K_{I}\int_{0}^{T}(\bar{Q_{est}}-\bar{Q_{Pr}})dt$$

where K_P_ and K_I_ were the proportional and integral gains, with the values 170 and 0.0001, respectively. The values were determined by using a critical proportioning method [17].

### 2.3. Parameters Setting for Physiological States

The NAC-FSL and CSC system performances were compared under hemodynamic perturbations. When the patient with an implantable LVAD switched from a resting position to the moving position, a series of hemodynamic parameters were observed to have altered. Emax, HR, and R_S_ represented the heart contraction, the heart rate, and the system circulation resistance of heart failure patients, respectively, and further details were referred to [17]. Table 2 describes the parameter settings of control systems at rest and motion. This paper simulated 30 cardiac cycles, out of which, the initial 15 cycles simulated the patient’s resting state during 15 s, 2.5 s simulated the state changing, while 16 to 30 cycles simulated the patient’s motion state in the next 7.5 s.

## 3. Results

**Figure 4:**
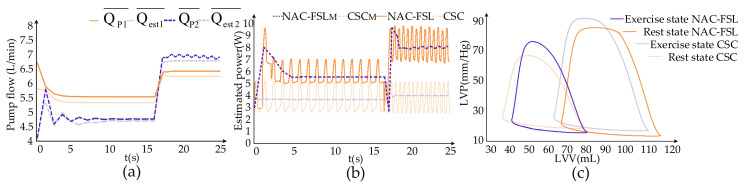
Effect of CSC control system and NAC-FSL control system on hemodynamic parameters from resting to exercise state. (**a**) The measured and estimated mean pump flow in two states. $\bar{Q_{P1}}$ and $\bar{Q_{P2}}$ represent the measured mean pump flows of CSC and NAC-FSL, respectively. $\bar{Q_{est1}}$ and $\bar{Q_{est2}}$ represent the estimated mean pump flows of CSC and NAC-FSL, respectively. The error between the estimated mean pump flow and the measured mean pump flow was compared. (**b**) The estimated power corresponding to the motor speed from resting to exercise. NAC-FSL_M_ represents the mean pump power at a variable speed and CSC_M_ represents the mean pump power at a fixed speed. (**c**) Changes in the P-V loops of the two control systems.

Figure 4 showed the effects of the control systems on hemodynamic parameters from the resting to exercise state. Rotational speed and pump flow are periodic parameters. Therefore, the mean rotational speed means the average value of rotational speed over its period and the mean pump flow refers to the average value over its period in this paper. From the resting to exercise state, the mean motor speed during a cycle of the CSC system remained constant at 2600 r/min. In contrast, the mean motor speed of the NAC-FSL system fluctuated at first and then remained at 2859 r/min at the resting state for the Frank–Starling mechanism. When changing to the exercise state, the mean rotational speed increased to 2945 r/min. As demonstrated in Figure 4a, $\bar{Q_{est1}}$ in the CSC system rose from 5.28 L/min to 6.33 L/min, with an increase of 1.05 L/min. The average error in the CSC system between $\bar{Q_{P1}}$ and $\bar{Q_{est1}}$ reached R = 0.991 by the algorithm. $\bar{Q_{est2}}$ in the NAC-FSL system increased from 4.6 L/min to 6.85 L/min, rising by 2.25 L/min from rest to exercise state, and the average error between $\bar{Q_{P2}}$ and $\bar{Q_{est2}}$ achieved R = 0.9552. The error between $\bar{Q_{P}}$ and $\bar{Q_{\mathrm{est}}}$ in the NAC-FSL system was smaller than that of the CSC system. When the control system changed from the resting to exercise state, the increase of average pump flow in the NAC-FSL system was higher than that in the CSC system. The higher pump flow helped the NAC-FSL system adapt to the blood flow required for state changes.

In this paper, the pump power (Pe) from a resting state to an exercise state is estimated under the control of two systems, according to the motor speed (ω). Equation (4) depicted the specific calculation for both the systems and the trend of Pe was indicated in Figure 4b. In the CSC system, Pe was between 2.94 and 5.01 W during both states. However, in the NAC-FSL system, Pe changed from 5.0 to 6.73 W during the resting state and 6.79 to 9.32 W during the exercise state. It means that in the NAC-FSL system, more power from the pump motor is needed to provide higher hydraulic performance, thus unloading the left ventricle more.

The ability to unload the left ventricle is an important indicator for evaluating the physiological control system. The P-V loop (the relationship between LAP and LVV) is always used to evaluate the ability to unload the control systems. In Figure 4c, the solid lines represent the P-V loop under the resting state, while the broken lines represent an exercise state. Comparing the P-V loops under both control systems, in the rest state, the P-V loop under the NAC-FSL shifts to the left distinctly against the CSC, with a stroke volume (SV) of 40.87 mL and ejection fraction (EF) of 52.19%. While in the exercise activity level, the scores of NAC-FSL and CSC system reach 47.75 mL vs. 41.53 mL and 42.39% vs. 37.49%, respectively. In general, the NAC-FSL control system unloads the left ventricle more than the CSC control system, with a higher EF close to normal levels.

The K value mentioned earlier was added to the controller to provide a means of altering preload sensitivity. The selection of an optimal scale factor (K) for patients with different levels of heart failure was explained in this paper in order to provide the appropriate pump flow. As suggested in Table 3, this paper approximately graded the degree of heart failure into severe heart failure (Emax = 0.5), moderate heart failure (Emax = 1.0), and mild heart failure (Emax = 1.5) [18].

**Figure 5:**
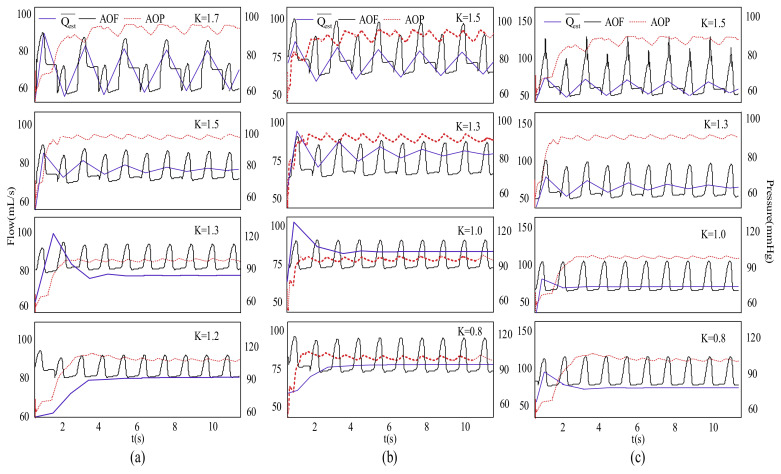
Changes in hemodynamic parameters as K values change for different degrees of heart failure. (**a**) Emax = 0.5, severe HF. (**b**) Emax = 1.0, moderate HF. (**c**) Emax = 1.5, mild HF. K: Scale factor. $\bar{Q_{est}}$: estimated average pump flow.

Figure 5 depicts the differences in hemodynamic parameters due to the changes in the value of K, thereby figuring out a more appropriate value of K for different degrees of heart failure. Figure 5a–c represent severe, moderate, and mild heart failures, respectively, and highlight the changes in aortic flow (AOF), aortic pressure (AOP), and $\bar{Q_{est}}$ for different K values. For a patient undergoing severe heart failure (Emax = 0.5), when K is less than or equal to 1.0, the control system will work out of the normal states and the data were over-adjusted. When 1.0 < K < 1.5, AOP, AOF, and $\bar{Q_{est}}$ tended to stabilize, but as the K value increased, the Frank–Starling-like control baseline CL_n_ tended to be flatter concerning smaller preload, resulting in a decrease in average pump flow. When K ≥ 1.7, severe oscillations were observed in $\bar{Q_{est}}$. Thus, when the patient undergoes severe heart failure, this paper recommends the K value to be about 1.3. When the patient experiences moderate heart failure, as indicated in Figure 5b, K ≤ 0.5, and the data are over-adjusted. For 0.5 < K < 1.3, AOP, AOF, and $\bar{Q_{est}}$ tend to stabilize. Similarly, $\bar{Q_{est}}$ gradually decreases with the increase in the K value. For K ≥ 1.3, the system data oscillates. When the patient experiences moderate heart failure, the K value of around 1.0 is recommended in this article. When the patient undergoes mild heart failure, in Figure 5c, and 0.8 < K < 1.5, AOP, AOF, and $\bar{Q_{est}}$ are relatively stable.

For K ≥ 1.5, the system would shock. In the case of mild heart failure, this paper suggests that the optimal K value was around 0.8.

## 4. Discussion

The NAC-FSL controller not only solves the postoperative complications in the traditional control system, which uses invasive sensors but also helps to prevent suction by the control curve construction of Frank–Starling. Since the NAC-FSL controller includes a flat slope at high preload, over-pumping at the high preload can be prevented.

Compared to CSC, the NAC-FSL evaluated in this study is able to synchronize the systemic and pulmonary flow irrespective of variations in the venous return by emulating the Starling mechanism of the native’s heart. When changing from a resting state to an exercise state, the increase of the average pump flow in NAC-FSL reached 2.25 L/min, which is higher than CSC. For the closed state of the aortic valve, the cardiac output was found to be equal to the pump flow. Therefore, it was concluded that the system under NAC-FSL could provide more cardiac output and meet the perfusion requirements under various physiological states. When changing from the resting to exercise state, the P-V loops under NAC-FSL shift to the left with a reduction in the area, and shift to the right with an increase in area under CSC. This means that the NAC-FSL system could unload the left ventricle effectively, so the left ventricle does less work. However, the CSC system under the exercise state could not help the effective unloading of the left ventricular. For matching the perfusion requirement in exercise, the left ventricle has to do more work, leading to the enlargement of the P-V loops.

Gaddum et al. (2014) utilized pulsatility-based controllers to imitate the native Starling flow sensitivity and proved that each hemodynamic parameter was superior to CSC [21]. In the controller system, pump pulsatility is a consequence of LV contraction, which is dependent on the LV preload. When severe heart failure occurs, the contraction of the left ventricle is not sufficient to provide a suitable pulsatility index for this control system. When the speed of the motor increases with the patient’s perfusion requirement, the unloading effect of the left ventricle becomes more obvious, and the contractility decreases accordingly.

To determine the patient’s working point OP_t_ in one cycle, the NAC-FSL control system proposed in this paper measured instantaneous preload instead of average preload. As a result, the system responded to the changes in the physiological state of the patient in time. Mahdi Mansouri et al. proposed a preload-based Frank–Starling system where they measured average preload to determine the patient’s working point and cost for at least two cycles and could not successfully respond to changes in the physiological state of the patient in time [18].

The NAC-FSL control system still had some problems that needed to be addressed. When setting the reference CVS to obtain a patient’s pathological preload, it is mandatory to obtain a large amount of information from the patient to fit into the patient’s cardiovascular model. Moreover, when the control system changes from resting to exercise state, the NAC-FSL has higher fluctuation than CSC. One reason may be the non-real-time control method. This issue needs to be solved in a future study.

In this study, the effect of K on control robustness and hemodynamics was investigated and appropriate K values were determined in the NAC-FSL control system. K value is always used to indicate preload sensitivity. The K value might be determined by the physiological states, the level of heart failure, and various other parameters. However, the appropriate way of selecting the K value remains uncertain. When designing and utilizing physiological control systems based on the Frank–Starling mechanism, the determination of the K value is crucial and needs to be solved. At each level of heart failure (mild, moderate, and severe), if the K is too small, the control system overshoots, and the motor speed goes out of control. As the K value increases, the waveform of parameters gradually becomes stable, but the error between $\bar{Q_{est}}$ and $\bar{Q_{P}}$ becomes larger, thereby compromising the accuracy of the controller. When K exceeds a predefined limit, the hemodynamic system parameters severely oscillate, and the unstable system is unable to adjust the LVAD motor speed to meet the perfusion requirement. By considering the stability, error, and overshoot of the control system, the optimal range of the K value under different degrees of heart failure was suggested in this paper. The K values in three grades of heart failure (mild, moderate, and severe) were recommended to be around 1.3, 1.0, and 0.8, respectively. In this study, overshoot and oscillation were observed for inappropriate K values. These phenomena must be avoided to prevent accidents while using a preload physiological control system based on the Frank–Starling-like mechanism. Further work will focus on the validation of the K value in in vitro and in vivo experiments.

## 5. Conclusions

The proposed NAC-FSL controller adjusts the rotational speed of the LVAD based on the physiological state of the patients to meet their perfusion requirements. This controller uses the Frank–Starling-like mechanism to track the optimal average pump flow. The merits and demerits of the NAC-FSL controller in different states were discussed. Suction was avoided by adopting the control baseline (CL_n_) that had a flat slope under high preload conditions. The NAC-FSL system could unload the left ventricle effectively and provide a greater pump flow and cardiac output with less error during the exercise state, as compared to the CSC system. Eventually, the K value in the NAC-FSL controller was optimized to meet the perfusion needs according to the hemodynamic parameters, which vary with different preload sensitivities (K values).

## Figures and Tables

**Table 1:** Setting of model parameters.

Parameters	Value	Physiological Meaning
f cell7 row 1 cell8 row 1	10.06	constant
g cell6 row 2 cell7 row 2 cell8 row 2	6.5-HCT × 3.25 × 10^−2^	Linearly related values to HCT
h cell6 row 3 cell7 row 3 cell8 row 3	HCT × 4.67 × 10^−3^–0.557	Linearly related values to HCT
i	0.009-HCT × 2.90 × 10^−4^	Linearly related values to HCT
j	0.0105	constant
k	5.5	constant
ρ	13,600	reference liquid density (kg/m^3^)
g	9.8	gravity acceleration (m/s^2^)
η	100%	efficiency of electrical power to hydraulic power
β	9.9025 × 10^−7^	pump parameter (mmHg/rpm^2^)

**Table 2:** Parameter settings for the physiological state.

Parameter	Rest State	Exercise State
Emax (mmHg/mL)	1.0	1.0
HR (bpm)	60	120
Rs (mmHg.s/mL)	1.2	0.5

**Table 3:** Comparison of controller sensitivity under different degrees of heart failure.

Emax	K	$\bar{Q_{\mathrm{est}}}(L/\min)$	$\bar{Q_{P}}\;(L/\min)$	Error	Stability
0.5	1.0	*	*	*	*
	1.2	5.0069	5.0591	1.68%	stable
	1.3	4.9773	4.9058	1.44%	stable
	1.5	4.7617	4.6595	2.15%	slight shock
	1.7	4.3598	4.5163	3.59%	shock
1.0	0.5	*	*	*	*
	0.8	5.1000	5.1933	1.87%	stable
	1.0	4.9686	4.8988	1.2%	stable
	1.3	4.7179	4.6247	1.4%	slight shock
	1.5	4.3560	4.4967	3.23%	shock
1.5	0.4	*	*	*	*
	0.8	4.9229	5.0477	2.24%	stable
	1.0	4.7588	4.8539	1.99%	stable
	1.3	4.3526	4.5904	5.46%	slight shock
	1.5	4.0980	4.4797	9.31%	shock

*—refers to a state of dysregulation and no valuable data.
